# TonEBP/NFAT5 expression is associated with cisplatin resistance and migration in macrophage-induced A549 cells

**DOI:** 10.1186/s12860-024-00502-y

**Published:** 2024-03-04

**Authors:** Hee Ju Song, Young Hwan Kim, Han Na Choi, Taehee Kim, Soo Jin Kim, Min Woong Kang, Sang Do Lee

**Affiliations:** 1 Department of Physiology, Chungnam National University College of Medicine, Daejeon, Republic of Korea; 2 Department of thoracic surgery, Chungnam National University School of Medicine, Daejeon, Republic of Korea

**Keywords:** A549, Cisplatin resistance, Migration, TonEBP/NFAT5

## Abstract

**Background:**

Macrophages promote angiogenesis, metastasis, and drug resistance in several cancers. Similarly, TonEBP/NFAT5 induces metastasis in renal carcinoma and colon cancer cells. However, the role of this transcription factor and that of macrophages in lung cancer cells remains unclear. Therefore, this study investigated the effects of macrophages and TonEBP/NFAT5 expression on cisplatin resistance and migration in A549 lung adenocarcinoma cells.

**Results:**

A549 cells were cultured alone or indirectly co-cultured with THP-1-derived macrophages using a transwell culture chamber. Cisplatin-induced cell death was markedly decreased and migration increased in co-cultured A549 cells. Macrophage-conditioned media (CM) showed a similar effect on drug resistance and migration. Cisplatin-induced apoptosis, DNA fragmentation, and cleaved apoptotic proteins PARP and caspase-3 were markedly reduced in macrophage CM-induced A549 cells. Here, ERK, p38, JNK, and NF-κB activities were increased by macrophage CM. Furthermore, the proteins involved in cisplatin resistance and cancer cell migration were identified using specific inhibitors of each protein. ERK and NF-κB inhibition considerably reduced cisplatin resistance. The increase in macrophage CM-induced migration was partially reduced by treatment with ERK, JNK, and NF-κB inhibitors. TonEBP/NFAT5 expression was increased by macrophages, resulting in increased cisplatin resistance, cell migration, and invasion. Moreover, RNAi-mediated knockdown of TonEBP/NFAT5 reduced cisplatin resistance, migration, and invasion in macrophage CM-induced A549 cells.

**Conclusions:**

These findings demonstrate that paracrine factors secreted from macrophages can change A549 cells, resulting in the induction of drug resistance against cisplatin and migration. In addition, the TonEBP/NFAT5 ratio, increased by macrophages, is an important regulator of the malignant transformation of cells.

**Supplementary Information:**

The online version contains supplementary material available at 10.1186/s12860-024-00502-y.

## Background

Lung cancer is the leading cause of cancer-related deaths worldwide, owing to complications affecting early diagnosis, poor cell reactivity to anticancer drugs, and metastasis [1–4]. Metastasis occurs due to cancer cell migration and invasion. Cancer cells spread throughout the body via the blood or lymph, and metastasis occurs as a result of penetration and proliferation of the migrated cancer cells into other tissues. As cancer cells migrate through the lymph, they often metastasize to tissues rich in blood vessels, such as the liver, lungs, and brain. Metastatic cancer cells have a high growth rate and low reactivity to anticancer drugs, thereby reducing patient survival rates [5–8]. Therefore, the genes that regulate cancer cell metastasis warrant extensive investigation.

Cancer cells are surrounded by the tumor microenvironment, which includes fibroblasts, leukocytes, the extracellular matrix, and macrophages [9]. Cancer and stromal cells in the tumor microenvironment secrete several cytokines, chemokines, and growth factors. Secreted soluble factors recruit macrophages from the bloodstream to the tumor periphery. Thus, the number of macrophages surrounding a tumor can predict cancer prognosis because they promote angiogenesis, metastasis, and drug resistance in several cancers. The infiltration level of macrophages into the tumor microenvironment is associated with poor prognosis in lung, breast, ovarian, stomach, and prostate cancers [10, 11].

Macrophages enhance the intravasation of cancer cells, which is strongly correlated with their migration through blood vessels [12]. Tumor-associated macrophages release C-C motif ligand 18 (CCL18) and the granulocyte-macrophage colony-stimulating factor (GM-CSF), which promote breast cancer invasion and metastasis [13, 14]. Furthermore, tumor-associated macrophages promote gemcitabine resistance in pancreatic cancer by modulating cytidine deaminase [15]. M2 type tumor-associated macrophages are associated with increased resistance to etoposide in lung and liver cancers [16]. In addition, tumor-associated macrophages promote cisplatin resistance in cancer stem or initiating cells by secreting milk fat globule-EGF factor 8 (MFG-E8) [17].

Cisplatin, a platinum-based drug, is one of the most widely used anticancer drugs [18, 19]. The efficacy of cisplatin depends on the type of cancer it is used to treat. When cisplatin efficacy against certain types of cancer is low, its concentration is increased to enhance its anticancer activity. However, cisplatin at higher concentrations causes adverse side effects, such as nephropathy, nausea, vomiting, and neurotoxicity [20–22]. Therefore, the dependency of cisplatin efficacy on cancer cell characteristics warrants further study. Further studies could facilitate the development of drugs that can increase cell reactivity and decrease resistance to cisplatin.

Tonicity-responsive enhancer binding protein/nuclear factor of activated T cells 5 (TonEBP/NFAT5) plays a key role in the maintenance of renal function. This transcription factor is widely expressed in most tissues and [23–25] is closely associated with the regulation of inflammatory responses [26, 27]. TonEBP/NFAT5 plays a key role in the differentiation of various cells, including adipocytes [28, 29]. It is associated with malignancy and induces metastasis by promoting S100A4 expression in renal carcinoma and colon cancer cells [30, 31]. Furthermore, integrin-induced transcriptional activity of TonEBP/NFAT5 causes cancer cell metastasis [32]. In addition, its expression is closely associated with decreased survival rate of patients with lung cancer [33]. Therefore, it is imperative to investigate the role of this transcription factor in lung cancer.

In this study, we investigated the signaling pathways that are activated by macrophages and required to promote cisplatin resistance and induce migration in lung cancer. Additionally, we investigated the relationship between TonEBP/NFAT5 expression and cisplatin resistance and migration in A549 cells.

## Results

### Cisplatin resistance and migration in A549 cells co-cultured with macrophages

**Fig. 1:**
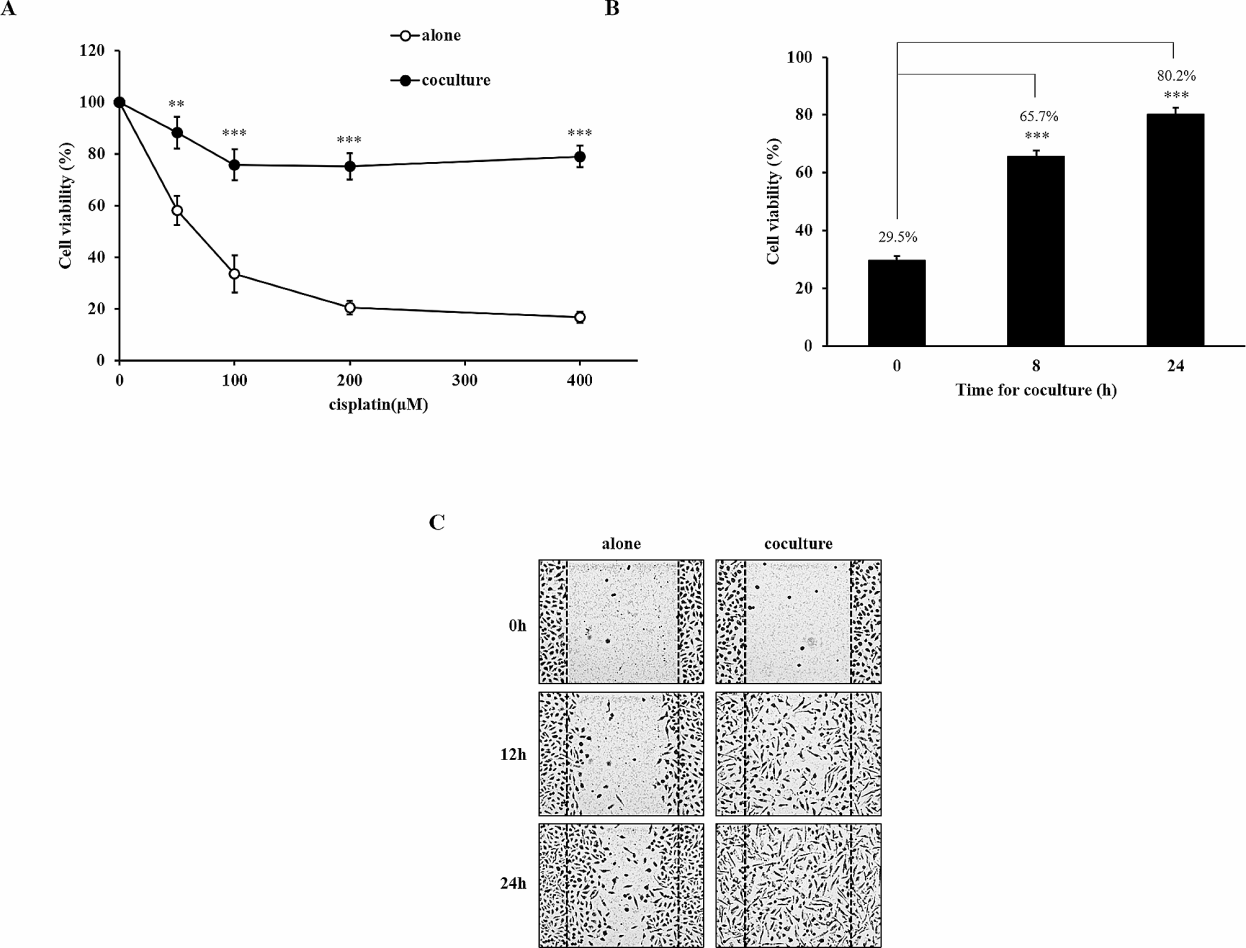
Effect of macrophage co-culture on cisplatin resistance and migration. (**A**) A549 cells were cultured alone or co-culutred with THP-1 derived macrophages for 24 h and then treated with various concentrations (0 ~ 400 µM) of cisplatin for 24 h. (**B**) A549 cells were cultured alone or co-culutred with THP-1 derived macrophages for 8 or 24 h and then treated with 200 µM cisplatin for 24 h. The cell viability was determined using MTT assay. (**C**) Migration ability was determined using a wound healing assay in A549 cells co-cultured with macrophages for 24 h. Data are average values ± SD of at least six independent experiments. ** *p* < 0.01, *** *p* < 0.001 (compared with alone and 0 h)

A549 lung adenocarcinoma cells were indirectly co-cultured with THP-1-derived macrophages in a Boyden chamber to investigate the effect of macrophages on cancer cells. The viability of A549 cells was 60%, 35%, and < 20% at 50 µM, 100 µM, and 200 µM cisplatin, respectively. In contrast, A549 cells co-cultured with macrophages for 24 h maintained a cell viability of 80% at all cisplatin concentrations, indicating that macrophages induced cisplatin resistance (Fig. 1A). The induction of cisplatin resistance by macrophages was significant from a co-culture time of 8 h (65.7%), and the most robust resistance was obtained at 24 h (80.2%) (Fig. 1B). Here, macrophages induced migration and cisplatin resistance in cancer cells. The wound-healing assay revealed that the migration of A549 cells co-cultured with macrophages was increased compared to that of cells that were not co-cultured (Fig. 1C).

### Induction of cisplatin resistance and migration by macrophage CM in A549 cells

**Fig. 2:**
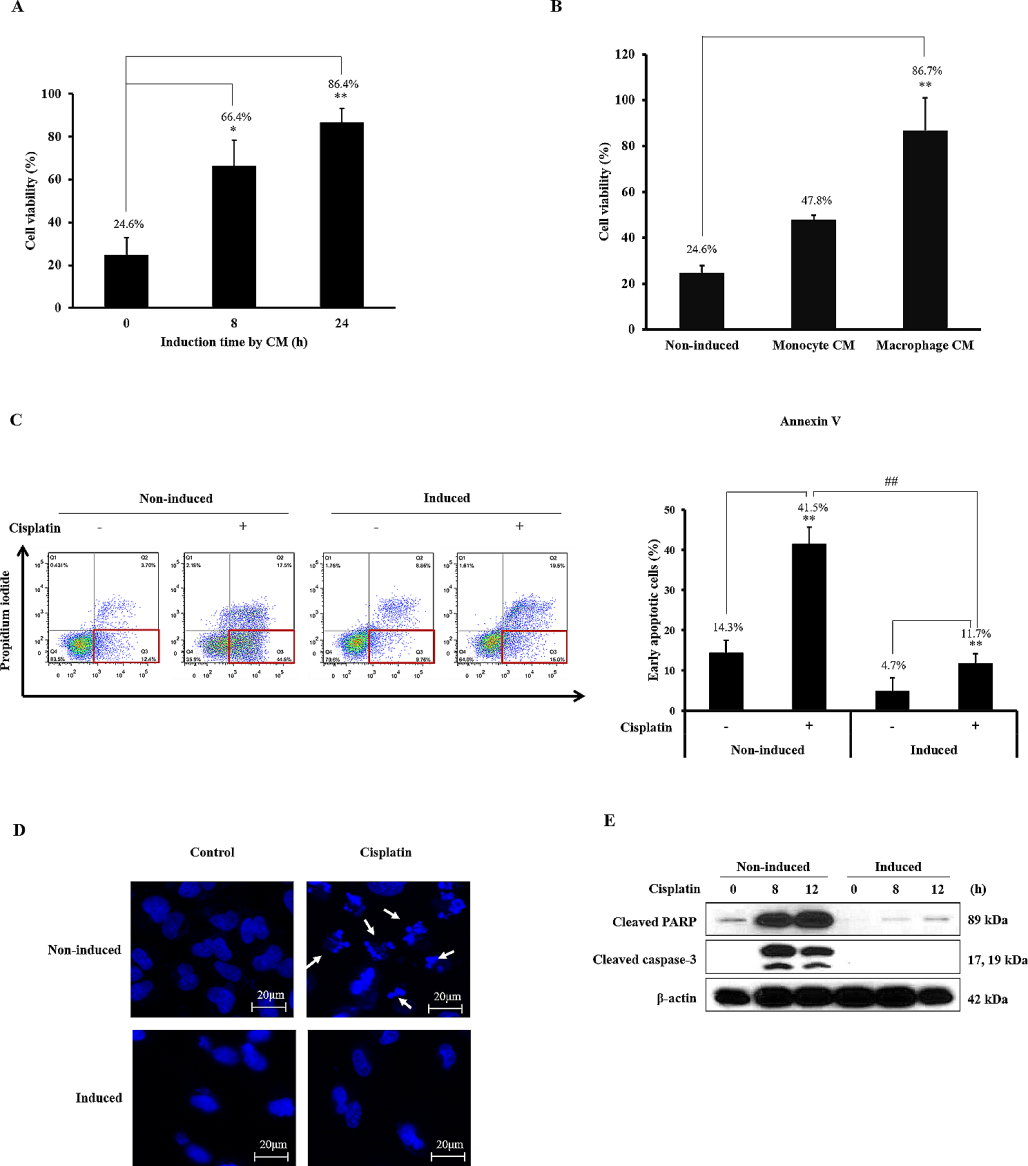
Induction of cisplatin resistance by macrophage CM. (**A**) A549 cells were incubated with macrophage conditioned media (CM) for 8 or 24 h and then treated with 200 µM cisplatin for 24 h. (**B**) A549 cells were incubated with monocyte or macrophage conditioned media (CM) for 24 h and then treated with 200 µM cisplatin for 24 h. Cell viability was evaluated using a MTT assay. (**C-E**) A549 cells were induced with macrophage CM and treated with 200 µM cisplatin for 8 h. Cisplatin induced early apoptotic cells were identified using Annexin V and PI staining. Cisplatin induced DNA fragmentation was identified using DAPI staining. Cleavage of apoptotic proteins, PARP and caspase-3 were determined by immunoblotting. Full-length blots are presented in Supplementary Fig. [1]. Data are average values ± SD of at least three independent experiments. * *p* < 0.05, ** *p* < 0.01 (compared with 0 h and no cisplatin-treated group), ## *p* < 0.01 (compared with CM-induced cisplatin-treated group)

In this study, indirect co-culture with macrophages indicated that soluble factors secreted from these effector cells might act on cancer cells to induce cisplatin resistance and migration. To confirm this, conditioned media (CM) was prepared from the culture supernatants of macrophages and used to treat A549 cells. Similar to the co-culture, this macrophage CM considerably increased cisplatin resistance (8 h:66.4%, 24 h:86.4%). In contrast, monocyte CM (47.8%) did not show any effect (Fig. 2A and B).

To confirm whether macrophage CM-induced cisplatin resistance was caused by apoptosis, early apoptotic cells were observed using Annexin-V staining. Early apoptotic cells increased when treated with cisplatin (41.5%). However, these apoptotic cells were substantially reduced in macrophage CM-induced cells (11.7%) (Fig. 2C). Thus, DNA fragmentation that occurs during apoptosis was subsequently investigated. DNA fragmentation was not observed in macrophage CM-induced cells, even after cisplatin treatment (Fig. 2D). In addition, the cleavage of PARP and caspase-3, which are apoptosis markers, was investigated. Cleaved PARP and caspase-3 levels, which were increased by cisplatin in non-induced cells, decreased in macrophage CM-induced cells (Fig. 2E).

**Fig. 3:**
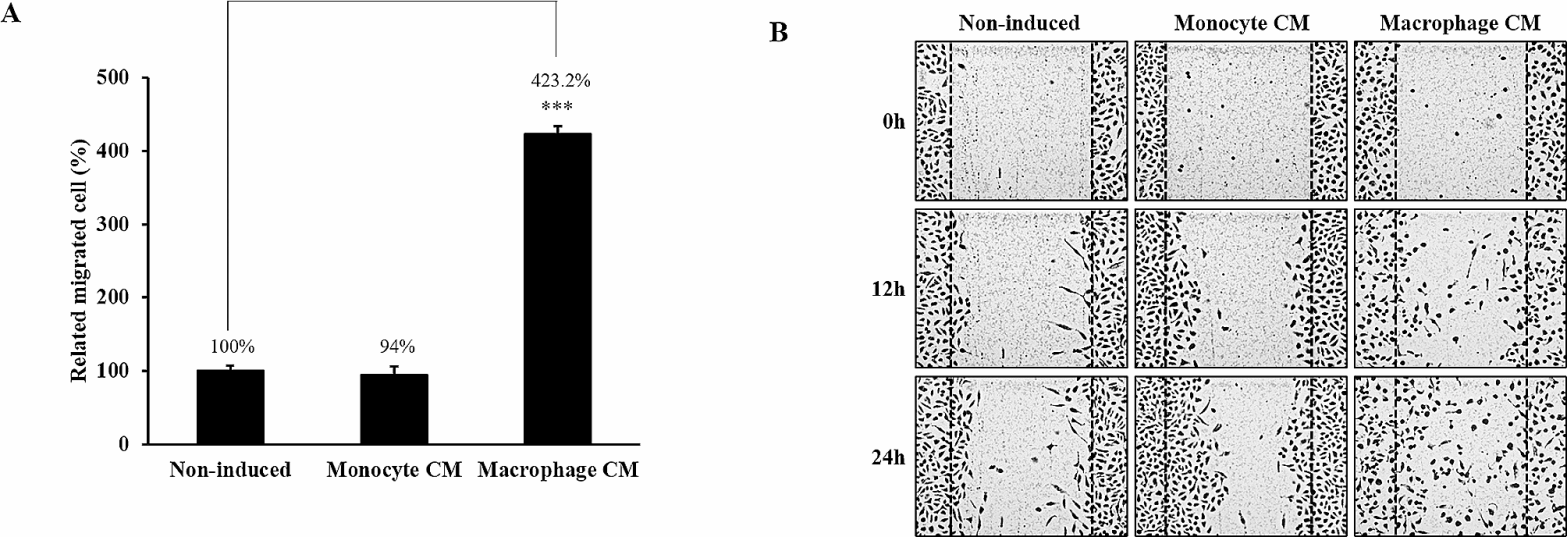
Induction of migration by macrophage CM. (**A**)A549 cells were incubated with monocyte or macrophage conditioned media (CM) for 24 h. Migration ability was determined using a wound healing assay. (**B**) Migrated cells were quantitated using a trans migration assay. Data are average values ± SD of at least five independent experiments. *** *p* < 0.001 (compared with Non-induced)

In a previous experiment, migration increased in A549 cells co-cultured with macrophages. This increase in migration was also induced in macrophage CM. In this study, increased cell migration was observed with macrophage CM in the transwell migration assay (423.2%). However, monocyte CM did not induce migration (94.0%) (Fig. 3A). The increase in migration by macrophage CM was also confirmed through the wound healing assay (Fig. 3B).

### Induction of cisplatin resistance and migration by macrophage CM in the NSCLC cell line

**Fig. 4:**
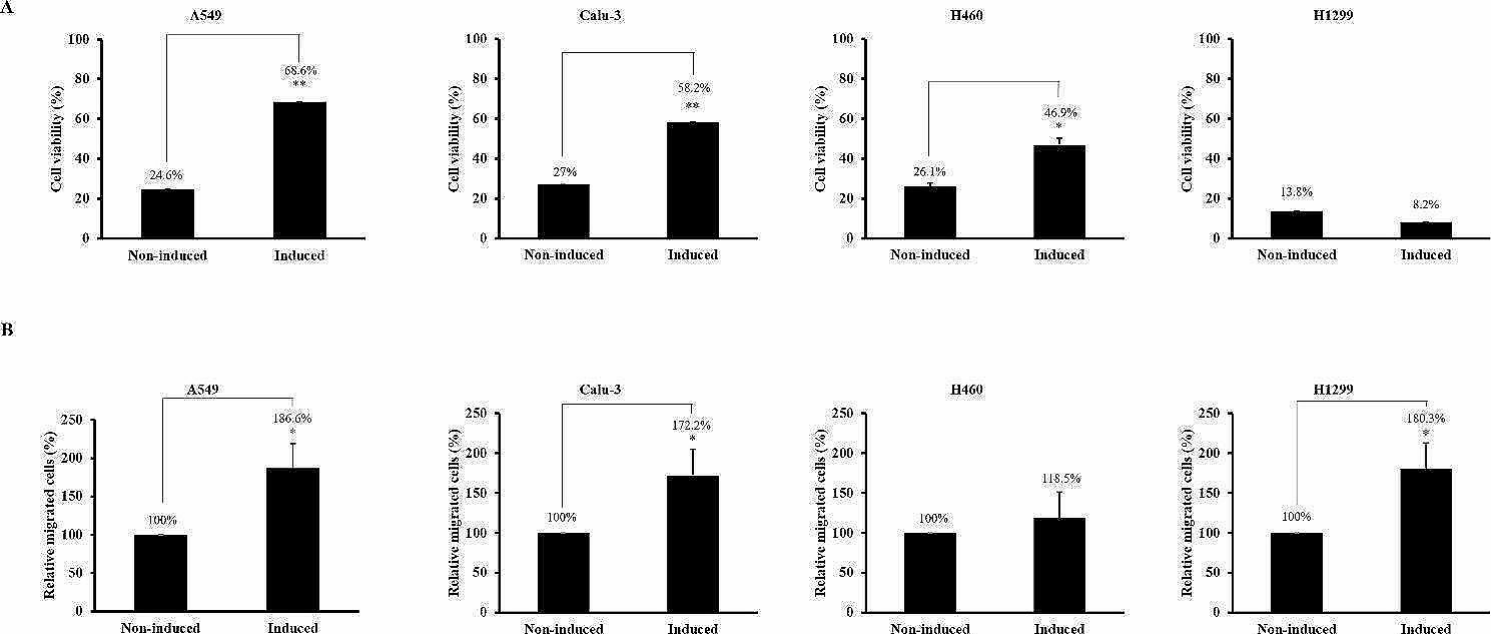
Macrophage CM induces cisplatin resistance and migration in NSCLC. (**A**) A549, Calu-3, H460, and H1299 cells were incubated with macrophage conditioned media (CM) for 24 h and then treated with 200 µM cisplatin for another 24 h. Cell viability was evaluated using a MTT assay. (**B**) Migrated cells were quantitated using a trans migration assay. Data are average values ± SD of at least three independent experiments. * *p* < 0.05, ** *p* < 0.01 (compared with Non-induced)

We investigated whether cisplatin resistance induced by macrophage CM was specifically induced in A549 cells or typically induced in non-small cell lung cancer (NSCLC) cell lines. Similar to A549 cells (68.6%), cisplatin resistance was significantly induced in Calu-3 (58.2%) and H460 (46.9%) cells following macrophage CM treatment. However, cisplatin resistance was not induced in H1299 cells (8.2%), even when cultured in macrophage CM (Fig. 4A). Migration induced by macrophage CM was also investigated in NSCLC cell lines. Migration was increased by macrophage CM only in Calu-3 (172.2%) and H1299 cells (118.5%), whereas it was not increased in H460 cells (180.3%) (Fig. 4B).

### Induction of cisplatin resistance and migration by macrophage CM via the ERK, JNK, and NF-κB pathways

**Fig. 5:**
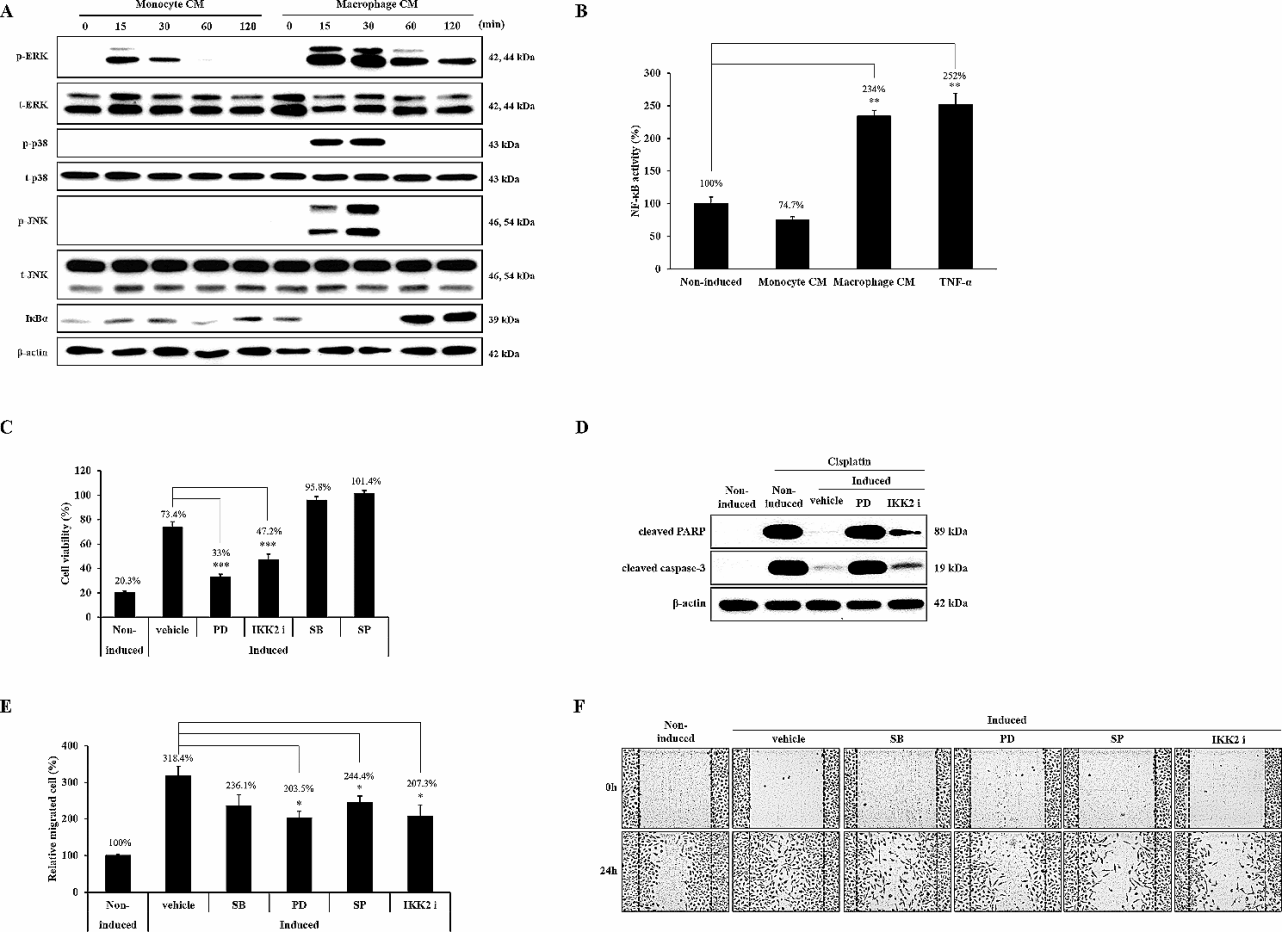
Signaling pathways are involved in macrophage-induced cisplatin resistance and migration. (**A**) A549 cells were incubated in monocyte or macrophage CM for the indicated times. Phosphorylation or the expression level of each protein was detected by western blotting using specific antibodies. The experiment was repeated with similar results. (**B**) A549 cells were transfected with an NF-κB-dependent luciferase expression plasmid and then incubated in CM or treated with 15 ng/ml TNF-α for 8 h. (**C**) A549 cells were induced with macrophage CM in the presence or absence of each inhibitor for 24 h, respectively. Viable cells in each condition were measured and compared with the corresponding control. (**D**) A549 cells were incubated with macrophage CM containing PD98059 or IKK2 inhibitor IV (IKK2 i). Cisplatin was added into each cell for 8 h. Whole cell lysates were subjected to western blotting. (**E**, **F**) A549 cells were treated with macrophage CM containing each inhibitor for 24 h. Migration ability was determined using a trans migration assay and a wound healing assay. The wound healing assay was repeated six times and representative images are shown. Full-length blots are presented in Supplementary Fig. (2). Data are average values ± SD of at least three independent experiments. * *p* < 0.05, ** *p* < 0.01, *** *p* < 0.001 (compared with Non-induced and vehicle)

Several cytokines have been reported to increase the survival and migration of cancer cells by activating the mitogen-activated protein kinase (MAPK) and NF-κB signaling pathways. Therefore, we examined the effect of macrophage CM on the activation of these pathways in A549 cells. As shown in Fig. 5A, phosphorylation of ERK, p38, and JNK and degradation of IκBα were markedly increased by macrophage CM but not by monocyte CM. The maximal activation of these pathways was observed after 15–30 min. Macrophage-induced phosphorylation of ERK was maintained for 2 h, and activation of p38, JNK, and NF-κB disappeared after 1 h. Furthermore, NF-κB promoter activity was considerably increased by incubating cells with macrophage CM for 8 h (234%). However, monocyte CM had no effect (74.7%) (Fig. 5B).

A549 cells were treated with specific inhibitors of each signaling pathway during induction with macrophage CM to assess the participation of ERK, p38, JNK, and NF-κB signaling in macrophage-induced cisplatin resistance. The specific inhibitor, PD98059, blocks ERK phosphorylation. SB203580 and SP600125 inhibit p38 and JNK activity, respectively. IKK2 inhibitor IV inhibits IKK2, an upstream factor of IκBα signaling. Inhibition of ERK (33%) or NF-κB (47.2%) activity substantially suppressed the induction of cisplatin resistance by macrophage CM. However, p38 (95.8%) and JNK (101.4%) inhibition did not affect cisplatin-induced cell death (Fig. 5C). To confirm the involvement of the ERK and NF-κB pathways in macrophage-induced cisplatin resistance, the effect of each inhibitor on cisplatin-induced apoptosis was examined. Cells were induced with macrophage CM containing ERK or NF-κB inhibitors and then exposed to cisplatin for 8 h. However, caspase-3 and PARP cleavage was completely inhibited in A549 cells incubated with macrophage CM. Inhibition of ERK activation during the incubation of A549 cells with macrophage CM induced levels of apoptosis similar to those observed in the control cells, whereas NF-κB inhibition did not (Fig. 5D).

To investigate the involvement of the MAPK and NF-κB signaling pathways in the enhanced migration, treatment with each inhibitor was performed during the induction of A549 cells with macrophage CM. The number of transmigrated cells was considerably reduced by inhibiting ERK and JNK activation, but not by the p38 inhibitor (Fig. 5E). Inhibition of macrophage-induced NF-κB signaling also suppressed the macrophage-induced transmigration ability of A549 cells (Fig. 5E). The participation of ERK, JNK, and NF-κB in macrophage-enhanced migration was confirmed using wound healing assays (Fig. 5F).

### Effect of TonEBP/NFAT5 expression of cisplatin resistance, migration, and invasion in macrophage-induced A549 cells

**Fig. 6:**
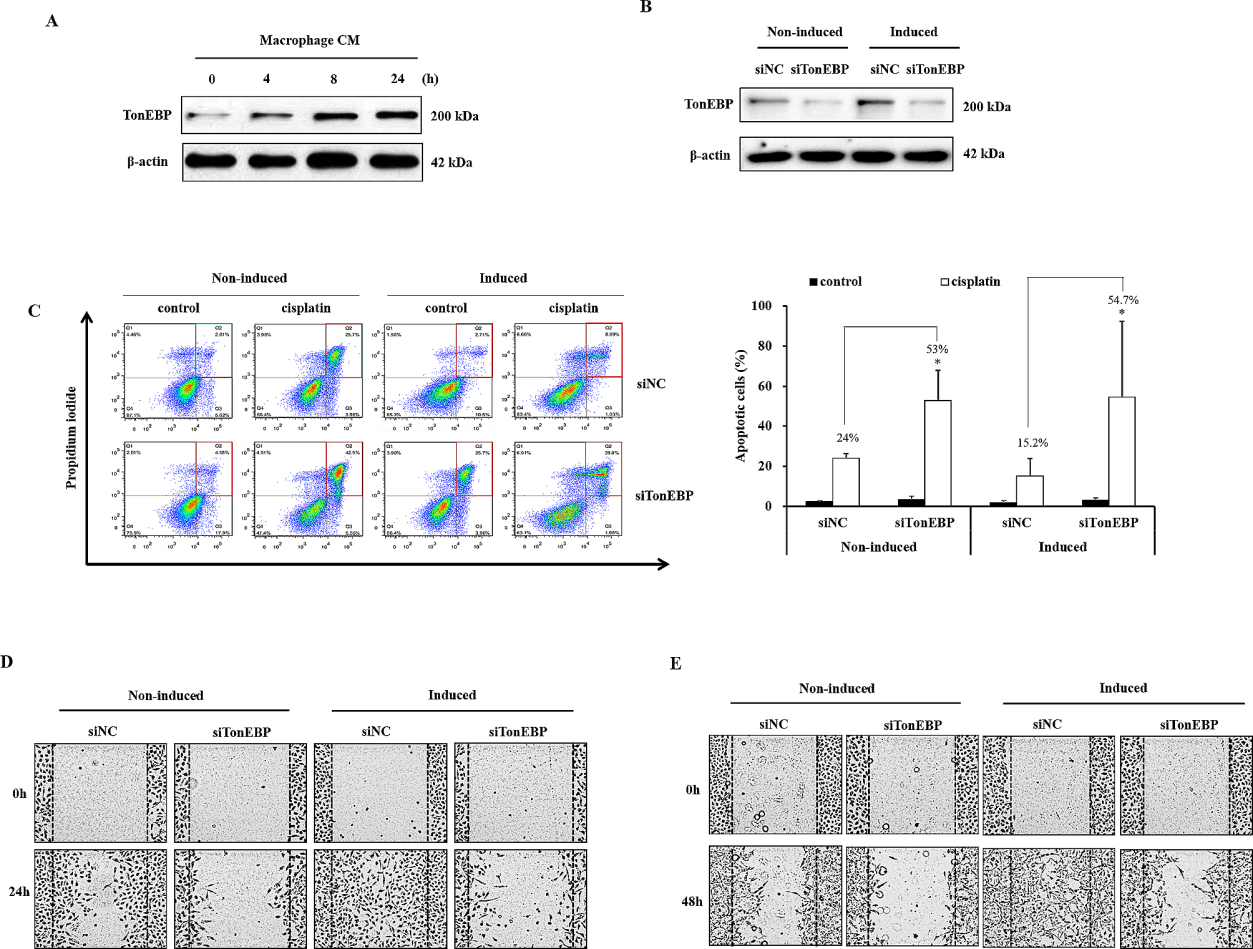
Effect of TonEBP expression of cisplatin resistance, migration and invasion in macrophage-induced A549 cells. (**A**) A549 cells were incubated in macrophage CM for the indicated times. The expression of TonEBP was analyzed by immunoblotting. (**B**) A549 cells were transfected with negative control or TonEBP siRNA. TonEBP knocked down cells were incubated in macrophage CM for 24 h. (**C**) Apoptosis was measured by Annexin-V assay. TonEBP knocked down cells were treated with 200 µM cisplatin for 8 h. Annexin-V and PI-stained cells were analyzed using flow cytometry. (**D-E**) TonEBP knocked down cells were incubated in macrophage CM for 24 h. Migration ability was determined using wound healing assay. Invasion ability was measured by adding 200 µL Matrigel after wounding. Full-length blots are presented in Supplementary Fig. (3). Data are average values ± SD of at least three independent experiments. * *p* < 0.05, (compared with siNC)

TonEBP/NFAT5 expression is closely associated with the survival rate of patients with lung cancer. Therefore, we confirmed that TonEBP/NFAT5 expression was affected by macrophages. TonEBP/NFAT5 expression increased after 4 h of incubation with macrophage CM and was the highest after 24 h of incubation (Fig. 6A). The increased TonEBP/NFAT5 expression in macrophage-conditioned medium was inhibited using siRNA (Fig. 6B).

To investigate the effects of TonEBP/NFAT5 expression on cisplatin resistance in macrophage CM-induced A549 cells, the cells were treated with cisplatin for 12 h after suppressing TonEBP/NFAT5 expression. In non-induced cells, apoptotic cells accounted for 24.0% of the total cell count after 200 µM cisplatin treatment for 12 h. In contrast, the percentage of apoptotic cells significantly increased to 53.0% in the cells with suppressed TonEBP/NFAT5 expression. When A549 cells induced by macrophage CM were treated with cisplatin, the percentage of apoptotic cells was 15.2%, which was lower than that of non-induced cells. When TonEBP/NFAT5 expression was inhibited in macrophage CM-induced A549 cells, the percentage of apoptotic cells significantly increased to 54.7% (Fig. 6C).

The migration ability of cells with suppressed TonEBP/NFAT5 expression was determined using wound healing assays to investigate the effect of TonEBP/NFAT5 on cell migration. Cells migrated in the control group when cultured 24 h after wounding, but not when TonEBP/NFAT5 expression was suppressed. However, TonEBP/NFAT5-suppressed cells did not show increased cell migration ability in macrophage CM (Fig. 6D). The effect of TonEBP/NFAT5 expression on cell invasion was investigated using Matrigel. After wounding, Matrigel was added to the upper layer, and invasion was measured by observing the cells infiltrated with Matrigel after 48 h. Invasion was observed in the control group but not after the suppression of TonEBP/NFAT5 expression. Furthermore, increased invasion by macrophages decreased when TonEBP/NFAT5 expression was suppressed (Fig. 6E).

## Discussion

Macrophages around cancer tissues are differentiated by various soluble factors secreted by cancer cells, which promote cancer growth and progression. They specifically affect the migration and invasion of cancer cells and the formation of new blood vessels during cancer progression [34]. In this study, cisplatin resistance occurred when induced by macrophages for at least 8 h in A549 cells. In addition, macrophage-induced A549 cells showed increased migration compared to non-induced A549 cells.

Induction of cisplatin resistance and cell migration by macrophages was not observed in A549 cells alone. Cisplatin resistance and cell migration were induced by macrophages in the Calu-3 and H460 NSCLC cell lines and in Calu-3 and H1299 cells, respectively. Cisplatin resistance or cell motility depends on the type of cell line because the receptors expressed in each cell are different [35, 36]. In addition, the signal transduction of cells that increase cisplatin resistance or cell motility may be different even if the cell receptors are the same [37, 38].

In human cancer tissues, necrosis occurs due to a low-oxygen environment and nutrient deficiency because new blood vessels do not reach the inside of the early cancer tissue [39]. Necrotic cells secrete various inflammatory factors that induce other macrophages. As a result, many macrophages surround cancer tissue, thereby accelerating cancer progression [40].

The involvement of the ERK, p38, JNK, and NF-κB pathways in macrophage-induced cisplatin resistance and migration of A549 cells was investigated. Inhibition of macrophage-stimulated ERK signaling considerably suppressed the induction of cisplatin resistance and protected cells from cisplatin-induced apoptosis. Similarly, suppression of the ERK, JNK, and NF-κB signaling pathways effectively inhibited macrophage-induced migration. These results suggest that macrophage-induced cisplatin resistance and migration are regulated by different mechanisms. Macrophages secrete not only interleukin family members, but also TNF, CXCL, and growth factors [41, 42]. In IL-6 knockout mice, tumor growth and formation were downregulated, and proliferation markers and MMP-9 were reduce [43]. In addition, IL-6 prevents etoposide- and melphalan-induced apoptosis in neuroblastoma [44]. Transforming growth factor-β (TGF-β) can increase the resistance of lung adenocarcinoma cells to gefitinib [45]. Moreover, EGF increases breast cancer cell migration in synergy with IL-1β [46]. Similarly, TNF-α enhances migration and metastasis through several signaling pathways [47]. Chemokines, including CXCL10, CXCL12, CX3CL1, CCL2, CCL5, CCL18, and CCL20, induce chemotaxis to promote cell migration and invasion in ovarian, lung, breast, and colorectal cancers [14, 48–52]. The involvement of these secretory factors in macrophage-induced cisplatin resistance or migration can be determined through gene targeting in macrophages or inhibition of the action of receptors in cancer cells. Cancer cells can become resistant to multiple drugs through several mechanisms. ATP-binding cassette (ABC) transporters such as ABCA, ABCB, ABCC, and ABCG mediate resistance to multiple drugs [53]. ABCB 1 and ABCC 3 are also regulated by ERK, which affects the response of mesothelioma cells to doxorubicin [54]. DNA repair pathways also confer resistance to chemotherapy in cancer cells [55]. Platinum chemotherapeutic agents, such as carboplatin, cisplatin, and oxaliplatin, induce DNA damage in cells. However, drug resistance by DNA repair proteins can occur. Excision repair cross-complementation group 1 (ERCC1) plays an important role in nucleotide excision repair [56]. Increased ERCC1 expression by ERK causes cisplatin resistance in melanoma [57]. In addition, breast cancer genes 1 and 2 (BRCA 1 and BRCA 2), which are DNA repair genes, are also regulated by ERK to modulate the response to doxorubicin [54]. As ERK regulates ABC transporters and DNA repair genes, the contribution of ERK downstream factors to macrophage-induced cisplatin resistance warrants further investigation.

Metastasis is a multifaceted process involving the migration and invasion of cancer cells through the degraded extracellular matrix (ECM). MMP-2 and MMP-9 are ECM-degrading enzymes, and u-PA activates this enzymatic cascade that can eventually initiate metastasis. Activation of these proteins is frequently detected in cancers [58–60]. MMP-9 and u-PA can be controlled by EGF through JNK/AP-1 signaling, consequently promoting cell migration [59]. Downregulation of ERK and NF-κB signaling pathways by gypenosides, used as folk medicine in China, inhibits activation of MMP-2, MMP-9, and u-PA. This results in the suppression of migration and invasion of human oral cancer cells [61]. Furthermore, the downregulation of MMP-2 and MMP-9 by inhibiting ERK and NF-κB activity reduces colon cancer cell migration and invasion [62]. Thus, the identification of the downstream target could help elucidate the relationship between ERK, JNK, and NF-κB signals and the macrophage-mediated migration effects on MMPs and u-PA.

A recent study showed that the survival rate of patients with lung cancer is linked to TonEBP/NFAT5 expression. The survival rate of patients with lung cancer was considerably lowered with increased TonEBP/NFAT5 expression [33]. Moreover, disease-free survival is closely linked to tumor cell proliferation, cell reactivity to anticancer drugs, and metastasis [63, 64]. Therefore, we investigated the relationship between macrophages and TonEBP/NFAT5 expression. TonEBP/NFAT5 expression increased with incubation time in the macrophage culture medium. In addition, the suppression of TonEBP/NFAT5 expression by macrophages decreased cell migration and invasion. These results showed that macrophages increased TonEBP/NFAT5 expression in cancer cells, thereby increasing cell mobility and invasiveness. A previous study focused on macrophage polarization by TonEBP/NFAT5 [65]. The results of this study showed that macrophages contribute to cell malignancy by regulating TonEBP/NFAT5 expression.

## Conclusions

In conclusion, paracrine factors secreted from macrophages can alter lung cancer A549 cells, resulting in the induction of drug resistance against cisplatin via ERK signaling. Contrastingly, ERK, JNK, and NF-κB signaling enhance migration. In addition, macrophages increase TonEBP/NFAT5 expression, which is an important regulator of the malignant transformation of lung cancer cells. Our findings could help develop therapies that inhibit the metastasis of cancer cells by regulating TonEBP/NFAT5 expression.

## Methods

### Cell culture and preparation of macrophage conditioned media

Human lung cancer A549 cells and monocytic leukemia THP-1 cells were obtained from the American Type Culture Collection (ATCC; Manassas, VA, USA). Both cell types were cultured in RPMI 1640 medium (Welgene, Daegu, South Korea) supplemented with antibiotics (Life Technologies, Carlsbad, CA, USA) and heat-inactivated 10% fetal bovine serum (FBS; GE Healthcare Life Sciences, Victoria, Australia) in a 5% CO2 atmosphere at 37 °C. The cells were routinely subcultured at approximately 80% confluence. Furthermore, THP-1 cells were treated with 30 nM phorbol myristate acetate (PMA; Sigma-Aldrich, Saint Louis, MO, USA) and cultured for 24 h to obtain macrophages. THP-1 cells that differentiated into macrophages were cultured in fresh culture medium for 24 h. Then, only the supernatant (macrophage-conditioned media) was collected and added to A549 cells.

### Measurement of cell viability

Cell viability was measured using the 3-(4,5-dimethylthiazole-2-yl)-2,5-diphenyltetrazolium bromide (MTT) assay. The cells were seeded in 24-well plates at a density of 40,000 cells/well. After induction with conditioned medium (CM) for indicated time, various concentrations of cisplatin were treated. Then, 20 µL MTT solution (5 mg/mL in phosphate-buffered saline; Sigma, St. Louis, MO, USA) was added to each well and the samples incubated at 37 °C for 3 h to obtain formazan crystals. The formazan crystals were dissolved in 500 µL dimethyl sulfoxide. Finally, cell viability was measured at 570 nm using a microplate reader (TECAN, Hombrechtikon, Switzerland).

### Measurement of apoptosis

Apoptosis was measured by staining cells with Annexin V-FITC (BD Biosciences, San Jose, CA, USA) and propidium iodide. Cells were plated at a density of 1 × 106 cells in a 100-mm dish. And incubated for 24 h in the presence of conditioned media the cells were treated with 200µM cisplatin for 8 h and then trypsinized for FACs analysis. The cells were then resuspended in 500 µL DPBS containing Annexin V-FITC and propidium iodide. The fluorescence intensity of each cell plate was measured using a BD FACSCalibur instrument (BD Biosciences), and the data were analyzed using FlowJo software (Tree Star Inc., Ashland, OR, USA).

### Assessment of apoptosis by DAPI staining

Cells were plated in 6-well plate. After 24 h, cells were treated with various concentrations of cisplatin for 8 and 12 h. Cells were fixed in methanol/DMSO (4:1) solution at 4 °C. Cells were washed with PBS and then stained with DAPI. Stained cells were observed under the fluorescence microscope (Zeiss, Oberkochen, Germany).

### Measurement of cell migration and invasion

Cell migration was measured using wound healing and transmigration assays. In the wound healing assay, cells were scraped using a micropipette tip to ensure disruption. Subsequently, images were captured at 100× magnification using the Nikon Eclipse Ti inverted microscope (Nikon, Tokyo, Japan). Cell invasion was then measured by adding 200 µL Matrigel (BD Biosciences), and the culture medium was replaced with a fresh culture solution. The transmigration assay was performed in a Boyden chamber with an 8-µm pore size and 24-well insert (Corning Inc., Corning, NY, USA). The treated cells were washed with DPBS and seeded into the upper chamber in serum-free RPMI medium. A medium containing 10% FBS was placed in the bottom well. The cells were stained with 30 µg/mL Hoechst 33,342. Then, images of stained cells on the membrane were captured using a fluorescence microscope (Zeiss, Oberkochen, Germany), and the cell count was determined using ImageJ software. Similar inserts coated with 100 µL Matrigel were used to measure cell invasion.

### Small interfering RNA (siRNA) transfection

Dicer-substrate siRNA targeting TonEBP/NFAT5 RNA was used to knockdown TonEBP/NFAT5 expression. The siRNA sequence used to target TonEBP/NFAT5 RNA was 5’-AUGUUUCUGAUGAAAACUCUUGGAA-3’, 3-‘UUUACAAAGACUACUUUUGAGAACCUU-5’. All siRNA was purchased from Integrated DNA Technologies (DS ScrambledNeg; Coralville, IA, USA). A549 cells were transfected with TonEBP/NFAT5 RNA or negative control siRNA for 48 h using the Lipofectamine™ 2000 transfection reagent (Invitrogen, Waltham, MA, USA) according to the manufacturer’s instructions.

### Western blot analysis

Cells were lysed using 200 µL radio-immunoprecipitation assay buffer (50 mM Tris-HCl, 150 mM NaCl, 0.1% NP-40, 0.5% sodium deoxycholate, and 0.1% sodium dodecyl sulfate, pH 8.0). Equal amounts of protein were separated using 10% sodium dodecyl sulfate-polyacrylamide gel electrophoresis (SDS-PAGE) and electro transferred onto nitrocellulose membranes. Membranes were blocked using Tris-buffered saline (20 mM Tris-HCl and 140 mM NaCl, pH 7.6) containing 0.1% Tween 20 and 5% non-fat dry milk at room temperature for 2 h. Subsequently, the membranes were incubated with primary antibodies at 4 °C overnight. They were then washed and incubated with horseradish peroxidase-conjugated secondary antibodies for 2 h. Signals were detected using a chemiluminescence assay kit (Thermo Fisher Scientific, Waltham, MA, USA).

### Statistical analysis

Statistical significance was determined using Tukey’s multiple comparison test (SigmaStat; Jandel, San Rafael, CA, USA). Data are expressed as mean ± standard error. Finally, statistical significance was set at *P* < 0.05.

### Electronic supplementary material

Below is the link to the electronic supplementary material.

Additional file 1 of “TonEBP/NFAT5 expression is associated with cisplatin resistance and migration in macrophage-induced A549 cells”

## Data Availability

Not applicable.
