# Supplementary material for: TonEBP/NFAT5 expression is associated with cisplatin resistance and migration in macrophage-induced A549 cells
Source: BMC Mol Cell Biol. 2024 Mar 4;25:6. doi: 10.1186/s12860-024-00502-y (PMC10913585; doi:10.1186/s12860-024-00502-y)

TonEBP/NFAT5 Expression is Associated with Cisplatin Resistance and Migration in Macrophage-Induced A549 Cells

Running title: TonEBP/NFAT5 regulates cisplatin resistance and migration in A549 cells

Hee Ju Song[1], Young Hwan Kim[1], Han Na Choi[1], Taehee Kim[1], Soo Jin Kim[1], Min Woong Kang[2] and Sang Do Lee[1]*

[1]Department of Physiology, Chungnam National University College of Medicine, Daejeon, Republic of Korea

[2]Department of thoracic surgery, Chungnam National University School of Medicine, Daejeon, Republic of Korea

# - Supplementary Figures

Supplementary Figure 1

Fig. 2E

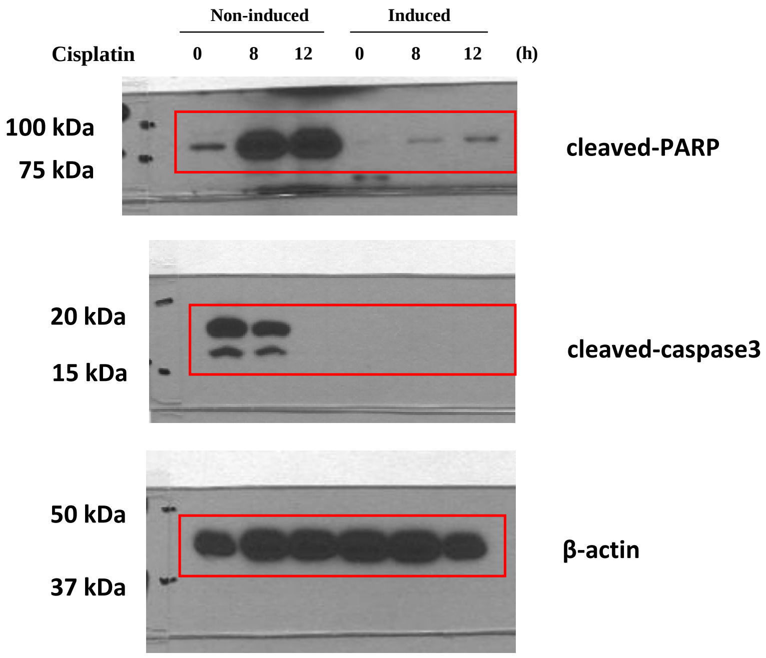

Supplementary Figure 2

Fig. 5A

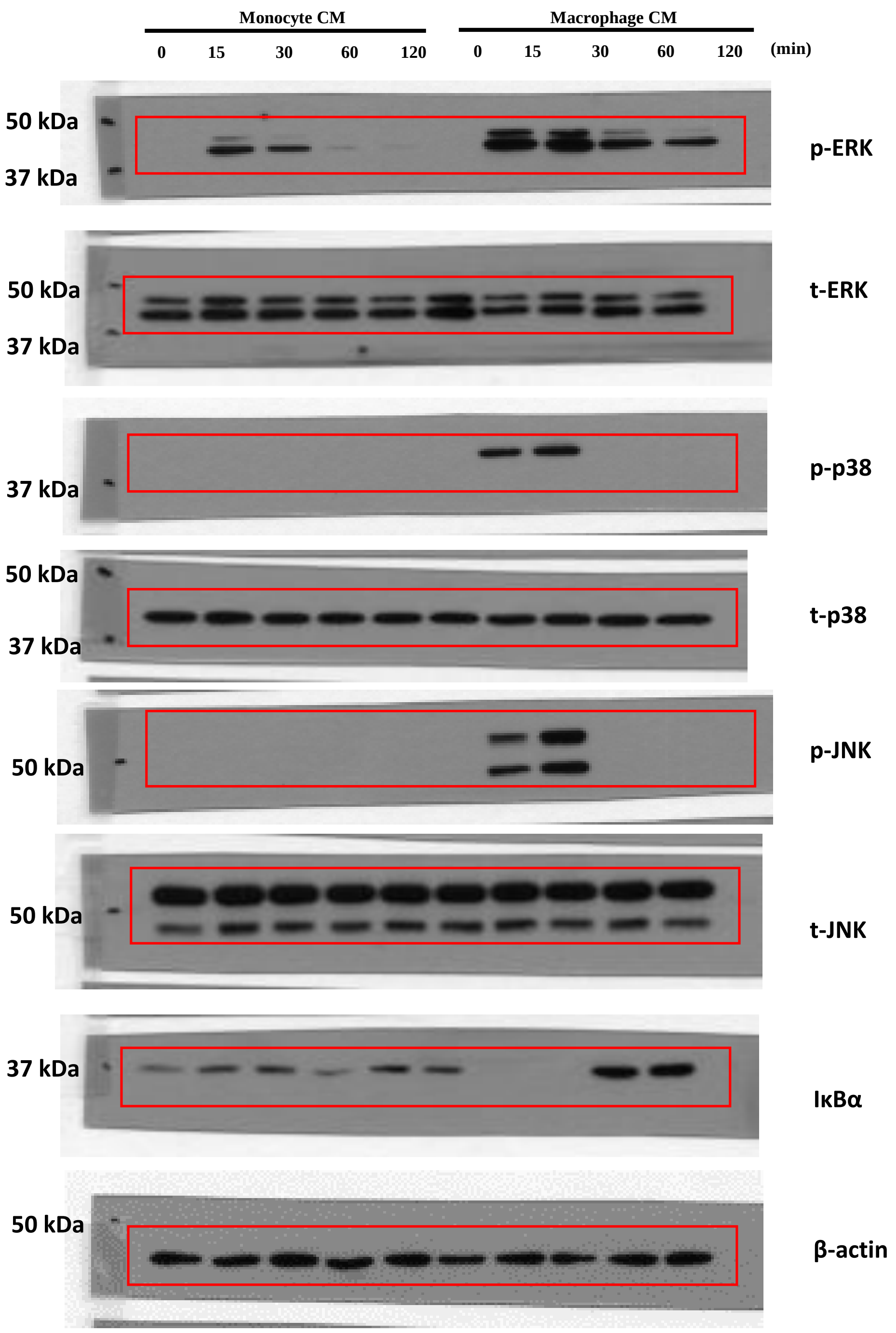

Fig. 5D

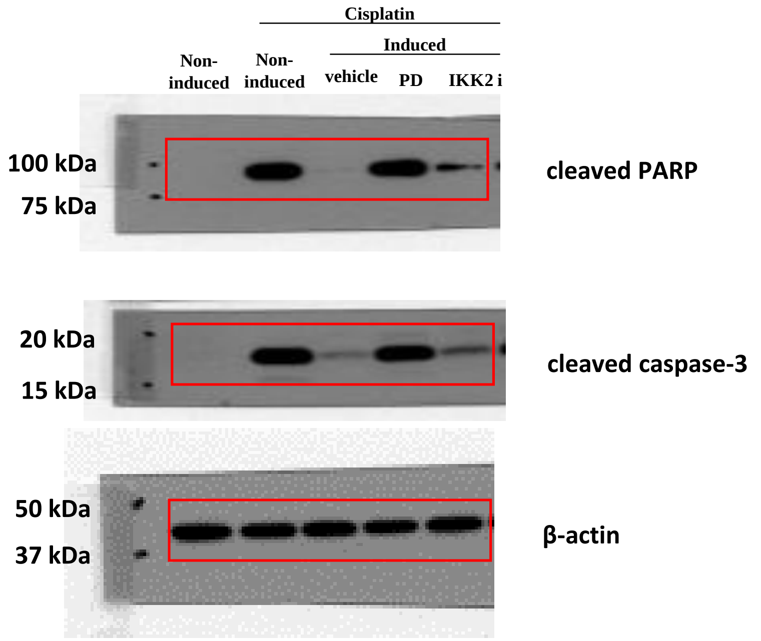

Supplementary Figure 3

Fig. 6A

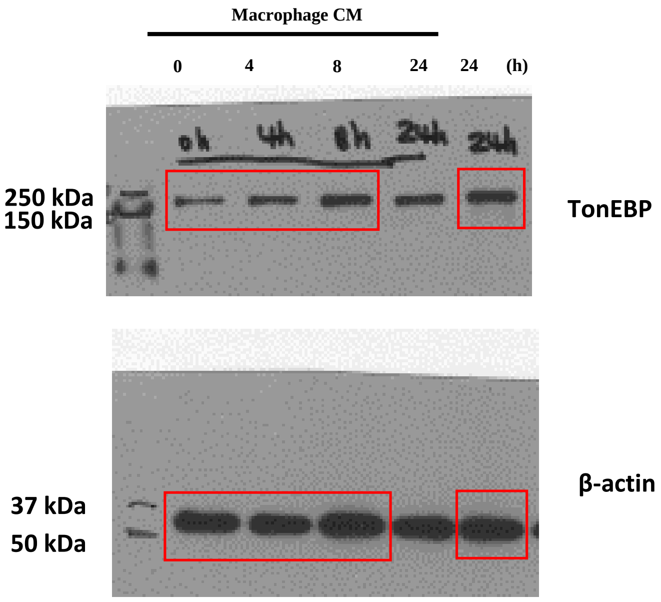

Fig. 6B

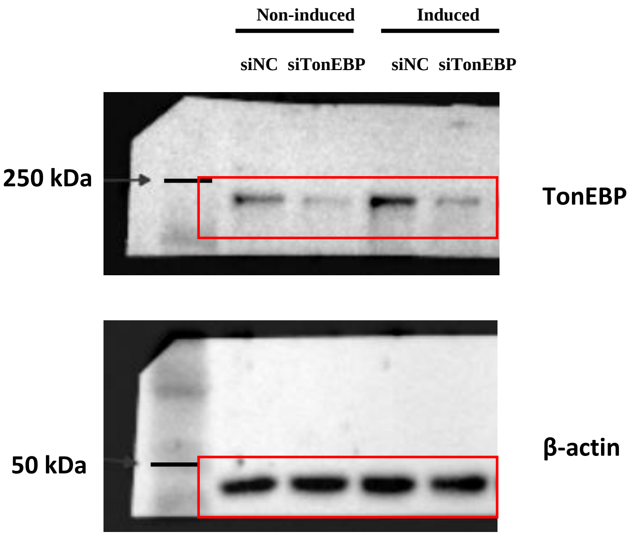

Supplement: Supplementary file 1 — Additional file 1 of “TonEBP/NFAT5 expression is associated with cisplatin resistance and migration in macrophage-induced A549 cells” [file 12860_2024_502_MOESM1_ESM.pdf]
